# Effect of *Carica papaya* on beta catenin and Wnt mRNA expression in human colon cancer (HT-29) cells in vitro

**DOI:** 10.6026/97320630018289

**Published:** 2022-03-31

**Authors:** Mohamed Rifaath, Preetha Santhakumar, J Selvaraj

**Affiliations:** 1 Saveetha Dental College and Hospitals, Saveetha Institute of Medical and Technical Sciences, Saveetha University, 162, Poonamallee High Road, Chennai- 600077, Tamil Nadu, India

**Keywords:** *Carica papaya*, beta catenin, Wnt, mRNA expression, human colon cancer (HT-29) cells, in vitro

## Abstract

Colon cancer is the third most frequent cancer in humans. *Carica papaya* leaves are vegetable foods consumed by most people around the world; it has potential as an anticancer. Therefore it is of interest to investigate the effect of *Carica papaya* on beta catenin and Wnt mRNA expression in human colon cancer (HT-29) cells in vitro. Human Colon cancer cell line (HT-29) was purchased from the National Centre for Cell Sciences, Pune, India. Cell viability test was done by MTT assay. Gene expression analysis was done by Real Time-PCR. The obtained data were analyzed statistically by one-way analysis of variance and Duncan's multiple range test with Graph Pad Prism version 5 to analyze the significance of individual variations among the control and experimental groups. The significance was considered at p<0.05 level in Duncan's test. *Carica papaya* caused a marked increase in cell death in a dose dependent manner. At the end of 48 hours, maximum inhibition was at 300 and 400 µg/ml. *Carica papaya* has significantly reduced the mRNA expression of Wnt and beta catenin (p<0.05). Data showed that *Carica papaya* leaf extract has anticancer activity on Colon cancer cell lines (HT-29).

## Background:

The medicinal plants have been used for many years to treat many diseases because of its therapeutics [1-3 - check with author]. Different parts of medicinal plant have numerous nutraceutical values and are enriched with proteins, carbohydrates, vitamins, fibre, potassium, calcium and other phytoconstituents which are having significant medicinal property. For many years herbal medicines are used and are still utilized in developing countries because the primary source of medical treatment. Plants are utilized in medicine for its natural antiseptic properties. Thus, research has developed into investigating the potential properties and uses of terrestrial plant extracts for the preparation of potential nanomaterial based drugs for diseases including cancer [4-7]. Natural active compounds derived from roots, barks, leaves or stems have been used in traditional medicine for many years within the treatment of various diseases, emphasizing the strong ought to determine their activity within the context of cancer treatment. *Carica papaya* belongs to the tiny family Caricaceae and is one among the main fruit crops cultivated in tropical and subtropical zones [8]. In traditional medicine, different parts of *Carica papaya* including its leaves, barks, roots, latex, fruit, flowers, and seeds have a good range of reputed medicinal application [9]. Experiments have shown that C. papaya possesses anthelmintic, antiprotozoan, antibacterial, antifungal, antioxidant, anti-inflammatory, antihypertensive, hypoglycemic and hypolipidemic, wound healing, antitumor, free-radical scavenging, antisickling, neuroprotective, diuretic, abortifacient, and antifertility activities [10]. The utility of herbal medicines for cancer treatment because they have no side effects and are cost efficient. Colon cancer may be a sort of cancer that begins within the intestine (colon). The colon is the final part of the alimentary canal. Carcinoma typically affects older adults, though it can happen at any age. It begins as small, noncancerous clumps of cells called polyps that form in the colon. Over time a number of these polyps can become colon cancers [11]. Wnt signaling is one among the key cascades regulating development and stemness, and has also been tightly related to cancer. The role of Wnt signaling in carcinogenesis has most prominently been described for colorectal cancer [12]. Beta-catenin is a multifunctional, 90 kD protein that contributes to cell development under normal physiological conditions. β-Catenin may be a crucial transcriptional consideration for Wnt signaling, and plays an important role in somatic cell renewal and organ regeneration. Besides, β-catenin promotes the progression of tumors via suppressing the T-cell responses [13-33]. Therefore, it is of interest to find the anticancer activity of the colon using *Carica papaya* leaf extract on Wnt mRNA and beta catenin mRNA expression.

## Materials and methods:

Dimethyl sulfoxide (DMSO), 3-(4,5-dimethylthiazol-2-yl)-2,5-diphenyltetrazolium bromide (MTT) were purchased from Sigma Chemical Pvt Ltd, USA. Trypsin-EDTA, fetal bovine serum (FBS), antibiotics-antimycotics, RPMI 1640 medium and phosphate buffered saline (PBS) were purchased from Gibco, Canada. (5,5,6,6-tetrachloro-1,1,3,3 –tetraethyl benzimidazolo carbocyanine iodide) and Real Time PCR kit was purchased TAKARA (Meadowvale Blvd, Mississauga, ON L5N 5S2, Canada).

## Cell lines and cell culture:

The Human colon cancer cell line (HT-29) was purchased from the National Centre for Cell Sciences (NCCS), Pune, India. Cells were cultured in DMEM medium (Thermo Fisher Scientific, CA, USA) containing 10% fetal bovine serum (Thermo Fisher Scientific, CA, USA), 100 U/ml penicillin and 100 µg/ml streptomycin (Thermo Fisher Scientific, CA, USA) at 37°C with 5% CO2.

## Cell viability by MTT assay:

Cell viability was assayed using a modified colorimetric technique that is based on the ability of live cells to convert MTT, a tetrazolium compound into purple formazan crystals by mitochondrial reductases (Mosmann, 1983). Briefly, the cells (1 x104/well) were exposed to different concentrations of *Carica papaya* extract (100-500µg/ml) with HT-29 cells for 48 h. At the end of the treatment, 100 µl of 0.5 mg/ml MTT solution was added to each well and incubated at 37°C for an hour. The formed crystals were dissolved in dimethyl sulfoxide (100 µl) and incubated in the dark for an hour. The intensity of the color developed was assayed using a Micro ELISA plate reader at 570 nm. The number of viable cells was expressed as the percentage of control cells cultured in serum-free medium. Cell viability in the control medium without any treatment was represented as 100%. The cell viability is calculated using the formula: % cell viability = [A570 nm of treated cells/A570 nm of control cells] x 100.

## Gene expression analysis by Real Time-PCR:

Samples from each group were submerged in 2 ml Trizol (Invitrogen, Carlsbad, CA, USA) for RNA extraction and stored at -80°C until further processed. cDNA synthesis was performed on 2 µg RNA in a 10 µl sample volume using Superscript II reverse transcriptase (Invitrogen) as recommended by the manufacturer. Real-time PCR array analysis was performed in a total volume of 20 µl including 1 µlcDNA, 10 µlqPCR Master Mix 2x (Takara, USA) and 9 µl ddH2O. Reactions were run on an CFX96 Touch Real-Time PCR Detection System (Bio-Rad, USA) using universal thermal cycling parameters (95°C for 5 min, 40 cycles of 15 sec at 95°C, 15 sec at 60°C and 20 sec at 72°C; followed by a melting curve: 5 sec at 95°C, 60 sec at 60°C and continued melting). For quality control purposes, melting curves were acquired for all samples. The specificity of the amplification product was determined by melting curve analysis for each primer pair. The data were analyzed by comparative CT method and the fold change is calculated by 2−ΔΔCT method described by Schmittgen and Livak (2008) using CFX Manager Version 2.1 (Bio Rad, USA).

## Statistical analysis:

The obtained data were analyzed statistically by one-way analysis of variance (ANOVA) and Duncan's multiple range test with a computer-based software (Graph Pad Prism version 5) to analyze the significance of individual variations among the control and experimental groups. The significance was considered at p<0.05 level in Duncan's test.

## Results:

### Effect of C. papaya on cell viability in HT-29 cells:

In the present study, *Carica papaya* extract significantly increased (p<0.05) inhibiting the growth of the colon cancer cells dose-dependently compared to untreated control cells. However, 300 to 400 µg/ml concentration of the extract showed maximum inhibition of the viability of the colon cancer cells suggesting that C.papaya induces apoptosis in HT-29 cells (Figure 1 - see PDF).

### Effect of C. papaya on Wnt mRNA expression in HT-29 cells:

In untreated control cells, Wnt mRNA expression was found to be significantly increased compared to treated control cells (p<0.05). Treatment with 300 and 400 µg/ml concentration of *Carica papaya* extract reduced the expression of Wnt mRNA (Figure 2 - see PDF).

### Effect of C. papaya on beta catenin mRNA expression in HT-29 cells:

In untreated control cells, beta catenin mRNA expression was found to be significantly increased compared to treated control cells (p<0.05). Treatment with 300 and 400 µg/ml concentration of *Carica papaya* extract reduced the expression of beta catenin mRNA (Figure 3 - see PDF).

## Discussion:

Carica papaya, an important plant, is well known for its medicinal properties [34 - check with author]. Different parts of the papaya plant are used traditionally in the treatment of disease conditions such as ulcers, eczema, asthma, diabetes, helminth infections and fever [35]. The present study was carried to investigate the role of *Carica papaya* on HT-29 cancer cell lines. In the present study, *Carica papaya* extract significantly inhibited the growth of the colon cancer cells in a dose-dependent manner when compared to untreated control cells. In 2002, Rahmat et al. had screened the anti proliferative activity of pure lycopene and of both juice and extracted lycopene from papaya and watermelon on human breast and cancer of the liver cell lines (two fruits with high lycopene contents) and reported that juice and pure lycopene caused necrobiosis within the cancer of the liver cell line HepG2 with the half maximal inhibitory concentration (IC50) of 20 mg/mL and 22.8 mg/mL, respectively [36 - check with author].

We have demonstrated that *Carica papaya* extract has a cytotoxicity effect. And this was similar to the studies by an author who has displayed anti-proliferative effects on tumor cells, promotion of Th1 type cytokine production, which has enhanced the cytotoxicity effects against tumor cells, and upregulates antitumor related genes in PBMC. Furthermore, it was reported that the active components of *Carica papaya* was responsible for its effect [37].

The activity of WNT/β-catenin in homeostasis and tissue development during a large spectrum of diseases has attracted biotech companies and medical attention. Its role in cancer formation and progression led to the research on search for anti-cancer agents targeting the Wnt/beta catenin pathway, and a variety of experiments have demonstrated that the inhibition of WNT pathway can affect the neoplastic survival and cell growth. In our study, the Caricapapaya has lowered Wnt/beta catenin expressions in colon cancer cell lines. And this was similarly studied by an author who showed plant products have lowered melanoma cancer cells by modulating Wnt/beta catenin signaling. Phytochemicals present in the plants are used in treatment of different types of cancers. Lots of plant derived compounds can modulate the Wnt/beta catenin signaling pathway [38]. The presence of the phytochemical in *Carica papaya* could be responsible for the anti-cancer effect through modulation of the Wnt/beta catenin pathway. The limitation of the study was that the study was only confined to two genes. In future, in vivo studies can be conducted to support the results of the study.

## Conclusion:

The present study has concluded that Carica papaya, with abundant bioactive phytochemicals, has the potential to be of use in combating colon carcinoma. However, there's an excellent need for more scientific investigations to enhance our understanding of how papaya may exert its anticancer effects. Further work is required to explore which bioactive compounds present in *Carica papaya* have anticancer effects and their mechanism of actions.
